# Mentalizing and self-other distinction in visual perspective taking: the analysis of temporal neural processing using high-density EEG

**DOI:** 10.3389/fnbeh.2023.1206011

**Published:** 2023-07-03

**Authors:** Vincent Rochas, Marie-Louise Montandon, Cristelle Rodriguez, François R. Herrmann, Ariel Eytan, Alan J. Pegna, Christoph M. Michel, Panteleimon Giannakopoulos

**Affiliations:** ^1^Functional Brain Mapping Laboratory, Department of Basic Neurosciences, University of Geneva, Geneva, Switzerland; ^2^Human Neuroscience Platform, Fondation Campus Biotech Geneva, Geneva, Switzerland; ^3^Department of Psychiatry, Faculty of Medicine, University of Geneva, Geneva, Switzerland; ^4^Department of Rehabilitation and Geriatrics, Geneva University Hospitals, University of Geneva, Geneva, Switzerland; ^5^Division of Institutional Measures, Medical Direction, Geneva University Hospitals, Geneva, Switzerland; ^6^School of Psychology, The University of Queensland, Brisbane, QLD, Australia

**Keywords:** automatic perspective-taking, brain rhythms, high density EEG, theory of mind, mentalization, inverse solution

## Abstract

This high density EEG report dissects the neural processing in the visual perspective taking using four experimental comparisons (Arrow, Avatar and Self, Other). Early activation differences occurred between the Avatar and the Arrow condition in primary visual pathways concomitantly with alpha and beta phase locked responses predominant in the Avatar condition. In later time points, brain activation was stronger for the Avatar condition in paracentral lobule of frontal lobe. When taking the other’s perspective, there was an increased recruitment of generators in the occipital and temporal lobes and later on in mentalizing and salience networks bilaterally before spreading to right frontal lobe subdivisions. Microstate analysis further supported late recruitment of the medial frontal gyrus and precentral lobule in this condition. Other perspective for the Avatar only showed a strong beta response located first in left occipito-temporal and right parietal areas, and later on in frontal lobes. Our EEG data support distinct brain processes for the Avatar condition with an increased recruitment of brain generators that progresses from primary visual areas to the anterior brain. Taking the other’s perspective needs an early recruitment of neural processors in posterior areas involved in theory of mind with later involvement of additional frontal generators.

## Introduction

Empathy refers to a complex construct that plays a key role in social adaptation and the quality of human relationships. Its affective component refers to the capacity of sharing emotions and responding immediately to the emotions of others; its cognitive component, commonly referred to as theory of mind (ToM) corresponds to the ability to understand other’s viewpoints, imputing desires and intentions (Decety and Jackson, 2004; Decety and Moriguchi, 2007; Young et al., 2007; Blair, 2008; de Waal, 2008). Recent lines of evidence suggested that theory of mind (ToM) is controlled by two distinct systems: one explicit that deliberately considers other’s thoughts and emotions and one implicit based on the automatic analysis of their viewpoints even when such analysis is irrelevant for task processing (Onishi and Baillargeon, 2005; Surian et al., 2007; Kovacs et al., 2010; Schneider et al., 2012). There is, in fact, a wide agreement that humans are able to engage in unconscious analysis of others’ mental states in the context of automatic perspective-taking (Samson et al., 2010; Low and Watts, 2013; Surtees et al., 2016; Schneider et al., 2017). This property may be of key importance for the mostly unconscious ascription of mental states needed for social interactions such as cooperating with colleagues and family members, thinking about others in their absence, and anticipating their emotional reactions. Consistent with this viewpoint, infants in the second year of life appear to represent others’ false beliefs when tested using implicit looking time measures (Onishi and Baillargeon, 2005; Kovacs et al., 2010), despite their poor performances on false belief tests that require explicit, verbal responses until 4 years of age (Wellman et al., 2001). Along the same line, patients with autism spectrum disorder show less evidence of implicit mentalizing than neurotypical individuals although both neurotypical and neuro-atypical individuals perform similarly on explicit verbal ToM tasks (Senju et al., 2009; Schneider et al., 2013).

Much of the debate surrounding implicit mentalizing has focused on the experimental results of the dot perspective-taking task (dPT), originally developed by Samson et al. (2010). In this task, participants are asked to count the number of dots on a screen. Importantly, an Avatar is also present on the screen when the dots are revealed and sees a number of dots that is either the same as (consistent trials) or less than the number of dots that the participant sees (inconsistent trials). Early fMRI observations showed an activation of the dorsolateral prefrontal and parietal cortices in dPT, mainly in inconsistent trials (i.e., the conflict between Self and Other perspectives) (Ramsey et al., 2013). In the same line, a domain-specific activation in several cortical areas such as the right temporo-parietal junction, ventral medial prefrontal cortex, and ventral precuneus was described in response to divergent other’s perspectives during self-perspective judgments (Schurz et al., 2015), but this position was later challenged (Santiesteban et al., 2017). The left TPJ/inferior parietal cortex as well as the bilateral inferior frontal gyrus (for inhibiting one’s own perspective) were consistently activated in visual perspective-taking paradigms. Using a large variety of neuroscientific methods (fMRI, near-infrared spectroscopy, transcranial direct current stimulation, and transcranial magnetic stimulation), the review of Bukowski (2018) reported a regular involvement of frontal lobe areas (dorsolateral PFC, posterior middle, and inferior frontal gyrus), dorsal precuneus, and TPJ, as well as the inferior parietal sulcus, inferior posterior temporal cortex, and superior cerebellum when judging the Other perspective. To date, there is still an ongoing theoretical debate of whether these brain activation patterns correspond to implicit mentalizing that would depend on the human nature of the Avatar or domain-general attention-orienting processes that would occur even when an Arrow replaces the Avatar form (for review see Furlanetto et al., 2016; Santiesteban et al., 2017; Cole and Millett, 2019; Westra et al., 2021).

One main limitation of these fMRI studies is their poor temporal resolution which does not provide information about the timing of the functional and neural processes in real-time involved in dPT. The use of classical EEG recordings makes it possible to monitor the neural responses to the task with high temporal precision. Previous contributions in this field remain scarce. Using Samson’s dPT task, McCleery et al. (2011) postulated that the temporoparietal cortex is involved in the perspective calculation (the distinction between Self and Other perspectives), whereas the right frontal cortex resolves the conflict between perspectives during response selection (McCleery et al., 2011). More recently, Ferguson et al. (2018) reported that the amplitudes of P100, P200, P300, and late frontal slow wave (LFSW) ERP components were reduced when a child Avatar was used in inconsistent trials (conflict between Self and Other perspectives), supporting an account where both mentalizing and directional processes modulate visual perspective-taking.

The present study aims at dissecting the different steps of neural processing in the dPT focusing on the distinction between mentalizing and non-mentalizing stimuli using: (A) exhaustive analysis of high-density EEG recordings at the surface and in the inverse space to define the brain sources of electrical activity, (B) microstate analysis that captures the dynamic activities of the large-scale brain networks, and (C) time-frequency decomposition to explore the dynamic changes in amplitude and phase of neural oscillations. Our main hypothesis is that, compared to Arrow (non-mentalizing stimulus), taking the Avatar (mentalizing stimulus) perspective implies the activation of neural generators in anterior cortical areas involved in emotional processing and ToM but also central executive areas. We also postulate that additional top-down and mentalization processes occur in fronto-parietal areas mainly when judging the Other Perspective for Avatar. In order to test our hypothesis, we included the comparison of EEG activation patterns using Arrow vs. Avatar according to the perspective taken (Self vs. Other-perspective). To simplify the experimental design and following the suggestion of Saether et al. (2021), we limited the present analysis to inconsistent trials (conflict between Self- and Other-perspective).

## Materials and methods

### Participants

The study was approved by the local Ethics Committee and all participants gave written informed consent prior to inclusion. All of the cases were recruited via advertisements in local newspapers and media. The final sample included 39 community-dwelling men (mean age = 31.6 years; SD = 11.4, range age 19–67 years). All participants performed a neurocognitive assessment. Subjects with a history of a chronic psychiatric disorder (psychosis or bipolar disorder), loss of consciousness lasting longer than 30 min, head injury or post-concussion symptoms, auditory or visual deficits, seizure and neurological disorders, and regular use of psychotropic medications were excluded.

### Dot perspective-taking task

**FIGURE 1:**
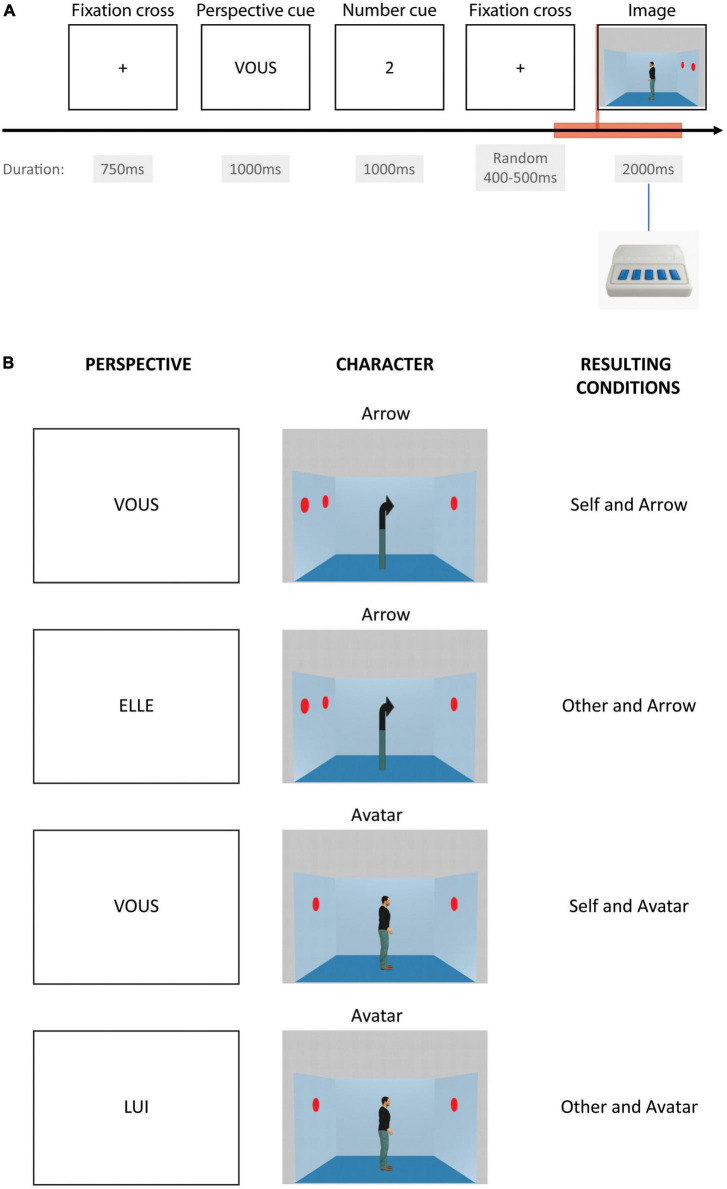
Perspective-taking task. The **(A)** displays one event time course. The red rectangle box shows the period of time taken into account for event-related analysis. The **(B)** displays the four different possible conditions depending on the instructed perspective cue in French (“VOUS” for the Self-perspective, “LUI” for the avatar, or “ELLE” for the arrow in case of the Other-perspective) and as a function of the character in the image (arrow or avatar).

The dot perspective-taking (dPT) task is derived from Samson et al. (2010) and has already been used in an EEG paradigm by Ferguson et al. (2018). The task used here was designed to match the EEG requirements of timing and repetitions. It is run with E-Prime 3.0 software (Psychology Software Tools, Pittsburgh, PA, USA) and displayed on a LED presentation screen. It consists of the presentation of a picture of a scene of an Avatar looking in one direction either left or rightward in a blue-squared room with a certain number of red dots painted on the walls (Figure 1). There is a total of zero to three dots displayed on each picture, distributed on the two-side walls of the represented room. The Avatar is shown as seeing between zero and two dots; no picture with the Avatar seeing 3 dots has been used in this version of the task. Except for a few trials using a no-dot picture, all the trials are inconsistent in terms of the number of dots seen by the participant and by the Avatar in the picture. A control set of pictures is equally used, displaying an Arrow instead of an Avatar. One trial starts with a fixation cross for 750 ms, then a perspective cue for 1000 ms instructing the perspective that has to be taken by the participant, i.e., himself or the Avatar-Arrow, followed by a number cue from zero to three displayed for 1000 ms as well. After a fixation cross, displayed for a random duration between 400 and 500 ms, the picture of the scene appears for 2000 ms. The participant’s task is to respond if the cued number corresponds to the number of dots actually seen from the perspective indicated. Correct or incorrect answers are delivered using a button press with the dominant hand after the scene is displayed. Responses are collected using an E-Prime Chronos box.

In total, 96 trials of the Arrow with Self-perspective, Arrow with Other-perspective, Avatar with Self-perspective, and Avatar with Other perspective are displayed. In addition, 48 filler trials displaying zero dots are also used to check for the attention of the participants and represent less demanding processing. For each condition, there is an equal proportion of correct and incorrect trials. Trials are displayed randomly in three distinct blocks of 144 trials in order to allow for little breaks during the task and preserve participants’ attention.

### EEG acquisition

Participants were seated in a dark and soundproofed Faraday cage in front of a LED presentation screen. High-density EEG was recorded during the task performance using an EGI 256 electrode Hydrocel Geodesic Sensor Net using a saline solution that was placed on the scalp of the participants and connected to an EGI GES 400 amplifier. The recording was done at a sampling frequency of 1000 Hz and with reference to the corresponding vertex channel (Cz electrode). The impedance of the electrodes was kept under 30 kOhms. Participants placed their chins on a chinrest situated at 80 cm from the screen and were instructed to keep still during the task.

### EEG pre-processing

Using the freely available software Cartool 3.91 (Brunet et al., 2011; Michel and Brunet, 2019),^1^ the channels corresponding to the cheeks and neck electrodes were removed resulting in 204 channels in total. The EEG was filtered with a DC removal (or 0 Hz high pass), a bandpass Butterworth filter from 1 to 80 Hz, and a Butterworth notch filter at 50 Hz and all possible harmonics. The recordings were reviewed by an experienced EEG analyst (VR), and the periods containing large movement artifacts and bad channels were discarded from further analysis. On average, 197 channels were used (mean channel number = 197.2; SD = 3.8). The data were then decomposed following an independent component analysis with Matlab using runICA from EEGlab. Based on their activation time courses and topographies, the resulting components identified as non-EEG (eye saccades and blinks, cardiac interference, and more rarely neck or jaw muscle tension) were subtracted from the data for further analysis. Using the software Cartool, the initially identified bad channels were reconstructed using 3D spline interpolation.

### Event-related analysis

The first series of analyses were focused on event-related processing locked on the scene picture onset. Epochs of 2000 ms were selected from −500 to + 1500 ms using the triggers for the four different conditions of the scene picture (i.e., Arrow with Self-perspective, Arrow with Other-perspective, Avatar with Self-perspective, and Avatar with Other-perspective). For each participant, the number of epochs was adjusted by randomly picking the lowest number to be equal between conditions and to have a comparable signal-to-noise ratio. On average, 80.9 epochs (min = 51 and max = 93) were taken into account for further analysis. The epochs were first averaged per participant per condition in order to compute ERPs (Event Related Potentials).

The clean EEG epochs of each participant for all four conditions were also used to compute event-related source reconstruction in Cartool (Michel and Brunet, 2019) in order to characterize at the source level the differences observed on the surface. The epoch data went through spatial filtering of the surface signal considering the position of the electrodes on the scalp. The employed inverse model was based on an MNI template head segmented into four shells (scalp, skull, CSF, and brain), 6008 solution points symmetrically distributed in the gray matter, and an EGI net model corresponding to the 204 remaining electrodes co-registered on the template scalp. A lead field was calculated for the segmented template head using Locally Spherical Model with Anatomical Constraints (LSMAC), an exact spherical equation in order to calculate a distributed linear inverse solution LORETA between the 204 electrodes and the 6008 solution points. An individual normalization using the background activity from the results of the inverse solution of the whole epoch data was used to estimate a baseline and a scaling factor for each solution point. We obtained individual normalized event-related source reconstructions in scalar values for the four different conditions.

### Microstate analysis

Brain generator activation taking place relatively late after the onset of the stimulus might be lost with a strict time-locked analysis as such processes usually occur with lower inter-individual consistency in time. In order to investigate these activations, we performed a microstate analysis from 500 to 1000 ms post-stimulus with no time constraint. We computed a segmentation of the grand averages in all participants during the above-mentioned time period for the four conditions separately using a k-means clustering technique adapted in Cartool, with 300 randomization trials taking into account the polarity of the topographies, rejecting the segments shorter or equal to 3 ms but without sequence constraint. The optimal cluster of topographies best explaining the data was defined according to a meta-criterion combining six criteria, i.e., Gamma, Silhouettes, Davies and Bouldin, Point-Biserial, Dunn, and Krzanowski-Lai indexes (Custo et al., 2017; Brechet et al., 2019). We fitted back the cluster topographies to each participant’s ERPs by condition to get the Global Explained Variance (GEV) of the different topographies as an index of representation at the individual level. The competitive fitting assigning the topography with the best correlation to the individual data was processed after smoothing with a window length of 6 ms and a Besag factor of 10 and rejecting for the segmentation the segments shorter or equal to 3 ms.

### Time-frequency decomposition

In order to refine the response to the stimulus picture in different brain oscillations, a decomposition of the signal was performed in the frequency domain over time. Time-frequency decomposition was computed using a fast Morlet transform in MATLAB 2018b on the selected clean epochs from −500 to + 1500 ms relative to the stimulus onset, reduced to absolute values, and averaged across epochs. Event-related spectral perturbation (ERSP) was computed by correcting time-frequency series by subtraction and division by the averaged baseline period from −400 to −100 ms (Neuper and Pfurtscheller, 2001). Inter-trial phase coherence (ITC) amplitudes were also estimated from time-frequency decomposition, reflecting the phase consistency across trials (Delorme and Makeig, 2004). ITC is the phase-locked oscillation part of the ERSP.

### Statistics

A multivariate two-way ANOVA on the percentage of correct responses and reaction times was performed in order to assess the effects of factor identity (Arrow vs. Avatar) and perspective (Self vs. Other) on the behavioral data. The surface ERPs were loaded in the all-channel randomization statistic toolbox RAGU (Koenig et al., 2011; Habermann et al., 2018 for details on statistical principles). Based on all channels and time points of all conditions, a multidimensional scale determined the disparity between participants and was used to define possible outliers, which were subsequently excluded from further analysis. A topographic consistency test, based on the comparison of the grand-mean global field power (GFP) of original data against the grand-mean GFP of shuffled maps, was conducted for each condition in order to define a period for which the neural activation across subjects remained consistent for further reliable ERP analysis. A two-by-two ANOVA, comparing differences among factors for original data against condition randomized data, with Arrow-Avatar and Self-Other-perspective within-subject factors was conducted on the global field power (GFP) for the period of time previously defined as showing topographic consistency across participants for all four conditions. A topographic two-by-two ANOVA with the same factor design was conducted similarly on topographies for the same period of time in order to reveal qualitative differences in neural processing distribution. In order to address the issue of false positives across time, the count of significant time points obtained in the original data was tested against the distribution of significant *p*-values for all randomization runs. The event-related source reconstructions for the four different conditions were tested between conditions using *t*-test statistics with false discovery rate (FDR) correction on the period of time previously defined as showing topographic consistency. For statistical analysis of the microstate results, the GEV of the different topographies was analyzed using two repeated measure ANOVA with identity Arrow-Avatar and Self-Other perspective within-subject factors. For both ERSP and ITC, cluster-based permutation tests were performed to explore condition-related differences in brain oscillations using a Matlab-based Fieldtrip function (Oostenveld et al., 2011).

## Results

### Data exclusion

One participant data set had a significantly higher multidimensional scale (RAGU) compared to the group distribution—thus showing low similarity among the entire data set. This participant’s ERPs contained strong residual alpha oscillations (a sign of low signal-to-noise ratio) and were in fact computed out of 46 epochs per condition only. This participant’s data set was considered an outlier and was discarded from further analysis. Considering the 39 remaining participants, ERPs were computed from 80.9 epochs on average (min = 51 and max = 93) out of 96 repetitions per condition.

### Behavioral performance

Participants performed very well in the task with an overall score of 94.9% (SD = 6.3%) of correct answers. The average reaction time was 852 ms (SD = 173 ms). Mean reaction times and the sum of the number of errors for Arrow and Avatar, Self and Other are summarized in Table 1. A multivariate two-way ANOVA on the percentage of correct responses and reaction times showed a multivariate effect of the factor identity (Arrow vs. Avatar) [*F*_(2,37)_ = 4.462, *p* = 0.018] with a solely univariate effect of the reaction time [*F*_(1,38)_ = 8.439, *p* = 0.006] which was significantly higher for Arrow than for Avatar condition. No effect of the factor perspective (Self vs. Other) reached significance.

**TABLE 1:** Demographic and behavioral data for the 39 included participants.

Variables	Mean	± SD	CI
Age (y)	31.59	11.25	28.1–35.1
Right-handedness	1.03	0.16	1–1.1
Education (y)	14.33	2.61	13.5–15.2
**Correctness (%)**			
Arrow self	94.25	6.93	92.1–96.4
Avatar self	94.90	6.88	92.7–97.1
Arrow other	95.28	6.26	93.3–97.2
Avatar other	95.03	6.29	93.1–97
**Reaction time (ms)**			
Arrow self	871.14	183.62	813.5–928.8
Avatar self	859.51	181.27	802.6–916.4
Arrow other	872.30	183.35	814.8–929.8
Avatar other	856.95	175.69	801.8–912.1

### Event-related analysis

#### Scalp level

**FIGURE 2:**
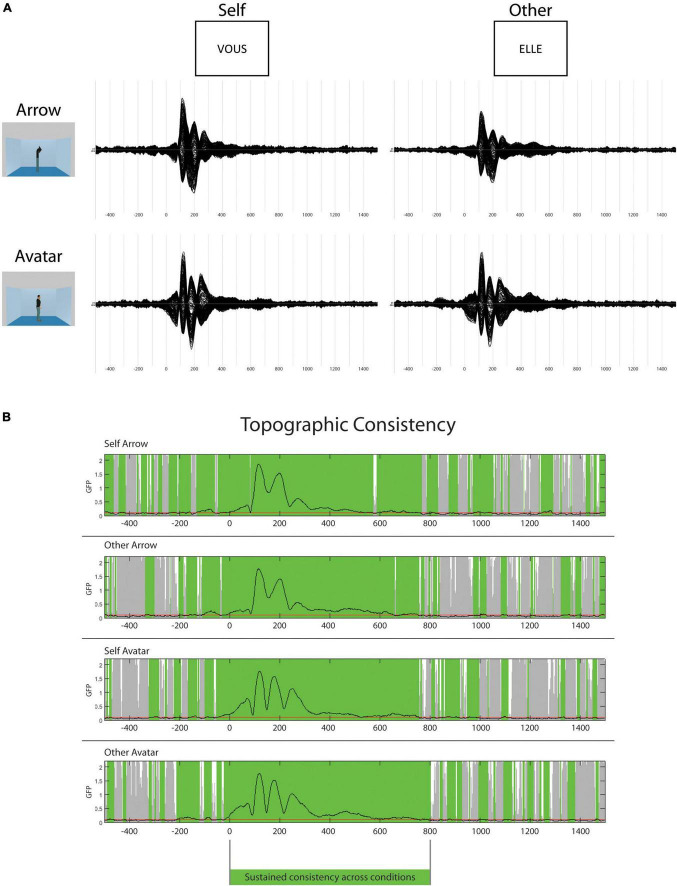
Event-related potentials (ERPs) and topography consistency. In the top panel **(A)**, group average ERPs for the four different conditions from –500 to 1500 ms (N: 39). In the lower panel **(B)**, the results of the topographic consistency test. The green overlay represents the periods of consistency across participants.

The topographic consistency test showed a period of sustained consistency for the ERPs in the four conditions from 0 to 800 ms (Figure 2). This period was then selected for the strict time-locked analysis. During the 300 ms post-stimulus, the GFP values varied with discontinued differences for Avatar and Arrow conditions. In contrast, consistently higher GFP values were observed in the Other vs. Self-conditions between 480 and 550 ms.

**FIGURE 3:**
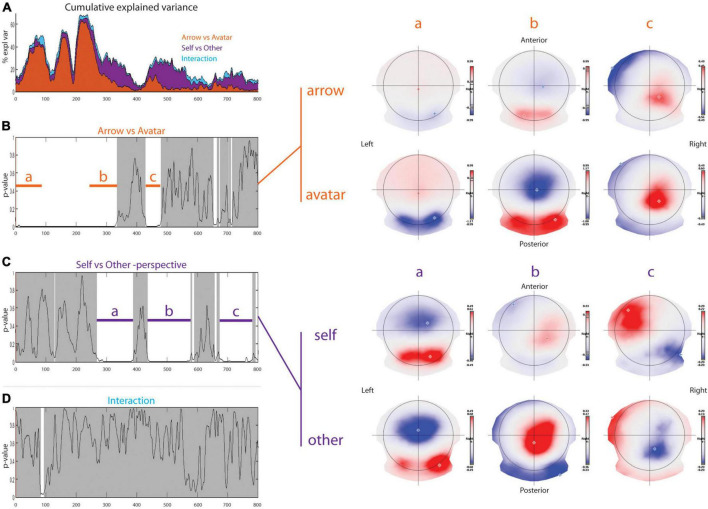
Topographic analysis. Results of the topographical ANOVA with Arrow-Avatar and Self-Other-perspective as within subject factors. In the first row on the left part **(A)**, the cumulative explained variance shows in which proportion each factor explains the data set. The *p*-value over time graphs for arrow-avatar **(B)** and self-other-perspective **(C)**, and their interaction **(D)** are depicted on the left part. The white overlay represents significant periods of time. On the right part, the average topographic representations for the two different factors illustrate the significant differences.

With respect to the Arrow vs. Avatar contrast (Figure 3), the TANOVA revealed early differences with a *p*-value distribution differing significantly from the random distribution (*p* = 0.0002) that can be divided into three-time windows. In the first 230 ms, there was a stronger rightward occipital pole negativity (opposed to fronto-parietal pole positivity) for the Avatar compared to the Arrow condition. This pattern persisted during the time period corresponding to the N75, P100, and N200 ERP components. These later displayed shorter latencies for the Avatar condition compared to the Arrow condition (Figure 2). Between 230 and 334 ms post-stimulus, there was a bilateral occipital positivity balanced with a sharp parietal negativity more pronounced for the Avatar compared to the Arrow condition. Finally, from 429 to 479 ms, there was a right parietal positivity more pronounced in the Avatar condition with a pole of negativity in the left frontal cortex that predominated in the Arrow condition. The Arrow vs. Avatar contrast allows for explaining up to 62% of the topographic variance during the first 460 ms post-stimulus.

With respect to the Self vs. Other contrast, topographic differences were found in three distinct time windows with a *p*-value distribution differing significantly from the random distribution (*p* = 0.0002). From 268 to 388 ms post-stimulus, the Other condition topography was associated with a marked positivity over the occipital lobe, mainly in the right hemisphere, and a strong negative pole centered on the parieto-frontal line. In the Self condition, there was a dipole axed postero-anteriorly mainly between the right occipital and right frontal lobes. From 436 to 577 ms the Other-perspective condition was associated with an inverted positivity-negativity in the same cortical areas. In contrast, the Self-perspective revealed a positive pole over the right parietal and a negative pole in the left frontal areas. Between 673 and 781 ms, there was a negative pole on the right parieto-occipital region and a positive pole on the left prefrontal edge for the Other-perspective with right occipital negativity and left frontal positivity for the Self-perspective. The Self vs. Other contrast explains almost 10% of the topographic variance at 250 ms with a subsequent increase to a peak of 25% at 500 ms.

The TANOVA revealed an interaction between Self-Other/Arrow-Avatar conditions that appeared significant only for the time window between 83 to 93 ms but revealed a non-significant amount of significant *p*-values (*p* = 0.7802).

#### Inverse solutions

**FIGURE 4:**
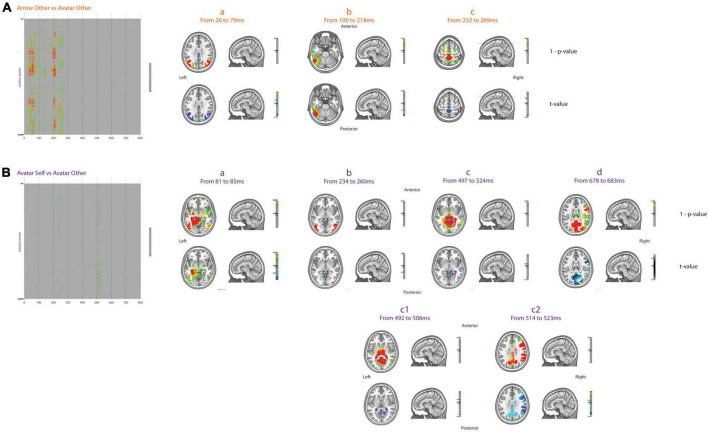
Inverse solutions. Results of *t*-test for two-by-two comparisons, i.e., arrow other vs. avatar other **(A)** and avatar Self vs. avatar other **(B)**, for the 6008 points across 800 ms after FDR correction. The green to red colors over the gray background displays the 1–*p*-values for significant comparisons only. On the right part, the average 1–*p*-values and *t*-values for different periods of interest are displayed in the MNI template head.

Considering the Other-perspective condition, the first differences in brain activation for the Arrow vs. Avatar contrast occurred in a large part of the posterior brain in favor of the Avatar from 26 to 79 ms (Figure 4A). These bilateral differences in brain activation were centered on the primary visual pathways between the inferior to middle occipital gyri on the left (BA19 and BA18) and middle temporal gyri (BA39) to inferior parietal lobule (BA7) on the right hemisphere. As already reported, this activation reflects the primary decoding of the picture in feature-oriented analysis (Kosakowski et al., 2022) and may be partly generated by the extrastriate body area, a region of the lateral occipito-temporal cortex responding to visual body stimuli (Downing et al., 2001; Pitcher et al., 2009). Stronger bilateral activity in the occipito-temporal regions, with a maximum average of differences from the left precuneus to the left inferior temporal gyrus (crossing angular gyrus, BA39, 19, 22, 27, 21, and fusiform gyrus) was observed for the Arrow condition between 100 and 218 ms. Consistent with the idea of a slower activation of neural generators in the Arrow condition, this latter was also associated with increased latencies of the classical early ERP components (N75, P100, and P200). Brain activation was subsequently stronger for the Avatar condition centered sagittally on the paracentral lobule of the frontal lobe (BA5) from 232 to 269 ms. Similar differences between the Avatar and Arrow conditions were also observed when considering the Self-perspective.

When considering the Avatar condition, the Self vs. Other differences in brain activation were first evident in the left parahippocampal and lingual gyri from 81 to 85 ms in favor of the Self-perspective. From 234 to 260 ms, the Self-perspective activation was predominant bilaterally in the middle occipital and middle temporal lobes (Figure 4B). From 492 to 506 ms, stronger brain activation was mainly observed bilaterally in the cuneus (BA19), precuneus, lingual gyri and posterior cingulate, and parahippocampus, as well as right angular gyrus for the Other-perspective. Between 514 and 523 ms, there was an additional activation of the right superior marginal gyrus and the inferior parietal lobule (BA40), and more anteriorly, the right pre-central gyrus (BA6) and the inferior frontal (BA9, 45) were all more pronounced for Other-perspective. Later on, between 678 and 683 ms, the Other-perspective was associated with increased activation in the precuneus (BA7) and cuneus (BA19) bilaterally as well as right frontal areas (BA6, 44, 45). Importantly, there were no significant differences between the Self and Other-perspective in the Arrow condition.

### Microstates

**FIGURE 5:**
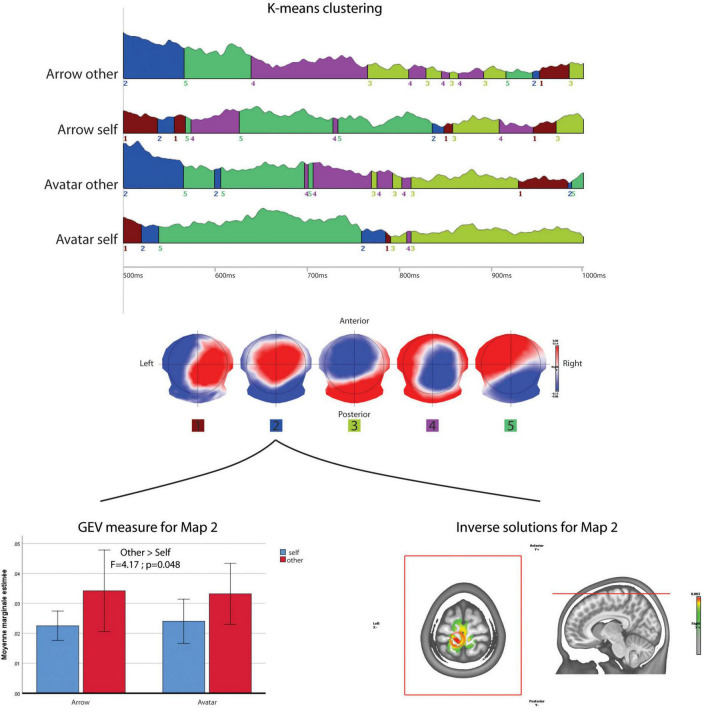
Microstate analysis. The results of segmentation in five maps of the 500 to 1000 ms periods of the four conditions. The lower row shows ANOVA results for the GEV of map 2 across the conditions and its projection in the inverse space.

After the K-mean decomposition of the 500 to 1000 ms period without time constrain, the clustering showed an optimal number of five maps that explain 78.31% of the topographical variance (Figure 5). Among these maps, map 2 with fronto-parietal positivity and occipital negativity had a higher Global Explained Variance for the Other compared to Self-perspective [*F*_(1,38)_ = 4.170; *p* = 0.048]. There was no significant effect according to the Arrow vs. Avatar factor. The projection of this map 2 in the inverse space localized a maximum of activity leftward in the medial frontal gyrus and pre-central lobule (BA6).

### Frequency response

#### Inter-trial coherence (ITC)

**FIGURE 6:**
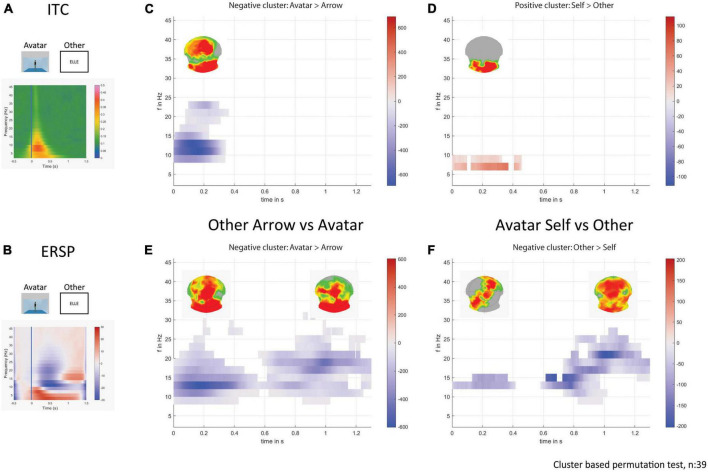
Time-frequency decomposition analysis. On the left, the plots show the average response for the avatar and other-perspective in ITC **(A)** and in ERSP **(B)**. On the right part, the plots display the statistically significant clusters in the sum of *t*-values across time and frequencies, and the corresponding *p*-values across electrodes on the above topographical maps for avatar over arrow **(C)** and self over other-perspective **(D)** in ITC. The plots display the statistically significant clusters in the sum of *t*-values across time and frequencies, and the corresponding *p*-values across electrodes on the above topographical maps for avatar over arrow **(E)** and other over self-perspective **(F)** in ERSP.

In both Self- and Other-perspective conditions, there was an increased ITC phase-locked to the stimulus in Avatar compared to Arrow conditions with a cluster between alpha and beta bands (8 to 24 Hz), with a maximum centered between 10 and 12 Hz and occurring between 0 and 350 ms (Other-perspective results presented in Figures 6A, C). This alpha and beta phase-locked response was present over the occipital lobes and medial frontal areas. The contrast between the Self- and Other-perspective revealed a cluster of higher theta synchrony between 6 and 8 Hz over the occipital lobes for the Self-perspective in the first 400 ms (Figures 6A, D) but only for the Avatar condition.

#### Event related spectral perturbation (ERSP)

In Other but not Self conditions there was an increased beta response around 12 Hz in the first 500 ms that persisted from 14 to 24 Hz after 600 ms. This beta rhythm activation was consistently more pronounced for the Avatar compared to the Arrow condition located in the occipital and medial fronto-parietal areas (Figures 6B, E). The Other-perspective showed a stronger beta response around 14 Hz compared to the Self-perspective in the first 400 ms located in the left occipito-temporal and right fronto-parietal areas followed by a larger band frequency response up to 26 Hz from 600 to 1300 ms (Figures 6B, F), mainly located in the frontal lobes. Such clusters of differentiation were only seen for the Avatar and not for the Arrow condition.

## Discussion

Using high-density EEG, the present study provides new insights into the complex cerebral mechanisms involved in dPT. Our data show that the temporal activation of brain generators depends both on the nature of the identity (Arrow vs. Avatar) and focus on the Self vs. Other perspective. Importantly, the two effects are largely independent since their interaction remains modest and is expressed only in a limited and very early post-stimulus time window (83 to 93 ms).

### Impact of the type of stimulus: non-mentalistic vs. mentalistic

Besides the increased activation of neural sources when treating mentalistic (Avatar) vs. non-mentalistic (Arrow) stimuli, our data document a mentalistic-related temporal shifting of brain generators from posterior visual areas to anterior frontal areas. Three sets of data support this statement. First, inverse space analysis revealed that the activation of the brain generators for the Avatar, but not Arrow, moves centrally in later time points (232 to 269 ms), from posterior occipital, middle temporal, and inferior parietal areas to the paracentral lobule of the frontal cortex. Second, the need for increased recruitment of brain generators in the Avatar vs. Arrow in early post-stimulus time points is also supported by our ITC analysis that revealed an increased alpha and beta phase-locked response in both Self and Other conditions over the occipital lobes and medio-frontal for the Avatar reflecting a systematic information transfer in the visual pathway and body representation (van Kerkoerle et al., 2014; Bastos et al., 2015). Third, the two ERSP bursts of frequency power unlocked to the stimulus in the beta band were also more pronounced for the Avatar with a strong and widespread beta rhythm response in occipital, parietal, and frontal areas between 400 and 1200 ms post-stimulus. However, unlike the ITC data, the increased recruitment of beta oscillations in Avatar compared to Arrow in the long-lasting ERP cluster was observed only in the Other condition when the participants needed to activate altercentric perspective-taking. These EEG observations agree with our recent fMRI study (contrasting Avatars and Arrows in inconsistent trials), reporting that in the presence of the Avatar, brain activation is observed not only in classical ToM areas such as the posterior cingulate cortex and precuneus but also in frontal lobe subdivisions and paracingulate gyrus (Montandon et al., 2023). Altogether, these results support the idea that mentalistic stimuli (Avatars) induce a distinct pattern of brain activation compared to non-mentalistic ones (Arrows), including parts of the posterior mentalizing networks but also anterior cognitive control-related areas.

These observations should be interpreted in conjunction with recent fMRI data and within the theoretical framework of the ongoing debate regarding mentalistic vs. non-mentalistic brain activation during the performance of dPT. Ramsey et al. first reported that in dPT, the automatic computation of the other’s perspectives takes place, which is independent of cognitive control (Ramsey et al., 2013). However, modifications of the dPT using transparent or opaque goggles were inconclusive with respect to spontaneous perspective-taking (Furlanetto et al., 2016; Cole and Millett, 2019). In a first fMRI study, Schurz et al. (2015) reported a spontaneous activation in the right temporoparietal junction (TPJ), ventromedial prefrontal cortex, and ventral precuneus during self-perspective judgments when using an Avatar (mentalistic) but not an Arrow (non-mentalistic control). This viewpoint has been challenged by neurostimulation reports, which show that transcranial magnetic stimulation of the right TPJ impairs performance on all self-perspective trials, indicating the predominance of attentional processes rather than implicit mentalization (Catmur et al., 2016; Santiesteban et al., 2017). With their excellent temporal resolution, our EEG data complete previous fMRI contributions and provide additional support to the distinction between mentalistic and non-mentalistic stimuli. The increase of alpha and beta oscillations time-locked to the stimulus for Avatar over the occipital lobes and medial frontal areas may be partly explained by the familiarity of this type of stimulus compared to Arrows. In studies of action observation, familiarity of the observer was associated with greater task-related beta power (Orgs et al., 2008; Di Nota et al., 2017).

Contrasting with the early brain reactivity for the Avatar condition, an increased recruitment of brain generators in the inferior temporal gyri was observed between 100 and 218 ms for the Arrow consistent with the idea of a slower activation of neural generators in the Arrow condition. This was also associated with increased latencies of the classical early ERP components (N75, P100, and P200). The marked differences in low-level features and stimulus categories between the two identities (Avatars are more complex but also more salient than Arrows for the participant) may be at the origin of the temporal differences in the activation of brain generators reported in this study. The ambiguity of the Arrow as a character could make its identification unnatural, weaker, and less consistent. Consequently, the reaction times for the Arrow condition are longer compared to the Avatar. In the same line, the Self-Other contrast in the inverse space may be detected only at a later time for Arrows, as demonstrated by our microstate analysis.

### Perspective taking: activation of brain generators

The combination of the EEG parameters studied in our study documents the complexity of the recruitment of neural sources in dPT. A predominant activation of areas involved in the rapid assessment of the visual scene and three-dimensional space analysis such as the parahippocampal gyrus, occipital, and lingual cortex (Epstein and Kanwisher, 1998) was first observed during 260 ms post-stimulus in the Self condition. These early activations of brain generators were followed by a strong involvement of the mentalizing [precuneus, posterior cingulate; (McCleery et al., 2011; Van Overwalle and Vandekerckhove, 2013)], and salience [angular gyrus (Arora et al., 2017)] and also frontoparietal executive networks [frontal BA 9, 6, 44, 45 (Ramsey et al., 2013)] in Other condition only starting from 500 ms. The activation of brain generators within the right frontal and temporoparietal areas for the Other-perspective is consistent with two previous EEG studies though with different types of analysis and alternate versions of the dPT (McCleery et al., 2011; Beck et al., 2018). The right inferior frontal gyrus activation was reported in late time periods (600–800 ms) by McCleery et al. (2011) in agreement with our microstate analysis that demonstrates the late recruitment of additional frontal generators (medial frontal and pre-central gyrus) involved in mentalization and mirror neuron activation (Coetzee and Monti, 2018) between 500 and 1000 ms. One could argue that the activation of the pre-central lobule could be an expression of the motor response and its preparation, but the Other vs. Self-difference observed here does not support the scenario of a simple motor command. Our ITC and ERSP data are consistent with the presence of complex processing with possible feedback mechanisms when assessing other’s perspective (Bastos et al., 2015). The phase-locked ITC analysis showed an early theta band recruitment in occipital areas for Self-perspective suggesting that rapid scene analysis and dot counting that relies on attentional processes are rapidly activated under this condition (Bastos et al., 2015). In contrast, the increased beta oscillations for Other condition was not time locked and occurred over occipito-parietal and later on frontal areas pointing to the additional brain effort needed for perspective selection and taking the other’s place in a ToM framework. An increase in beta power has been associated with both top-down processing of sensory information and task prioritization, two cognitive dimensions involved in altercentric judgment (Richter et al., 2018; Liegel et al., 2022). Some recent contributions pointed to the role of beta oscillations in mentalization processes both in clinical and non-clinical samples (Soto-Icaza et al., 2019; Mossad et al., 2022). The increased beta power in late time points observed in Other conditions for Avatar further supports a specific role for these oscillations in mentalization processes.

### Strengths and limitations

The present findings are based on high-density EEG and include topographic analysis, inverse space solutions, microstates, and oscillation analysis, both phase and non-phase locked to the stimulus. As such, it provides an overall view of the temporal and spatial activation of brain generators involved in dPT. However, there are several limitations. First, all of our cases were socially integrated young men without a history of criminal convictions and substance abuse who were initially recruited as part of a study focusing on psychopathy in the context of forensic psychiatry. The absence of women alone makes it difficult to generalize our results to the general population. Also, the careful exclusion of neurological and psychiatric disorders, regular use of psychotropics, as well as scores of all of the cognitive and emotional variables within the normal range limit the generalizability of our observations. Second, visual perspective-taking is one of the facets involved in social cognition. For some authors, the unconscious impact of other’s divergent viewpoints when we focus on our own visual experience is mostly driven by the activation of self-other distinction, self-updating via integration of self-relevant information, and central executive functions and mirroring (Bukowski, 2018; Alcala-Lopez et al., 2019). Our data did not address the correlations between these cognitive dimensions and EEG activation patterns. Third, the present findings focus on Samson’s dPT which is based on the judgment of visually presented situations and requires attribution of transient mental states without the need for decoupling representations (dot arrangements and not only dot numbers), propositional content, and overt action. As such, this paradigm cannot be seen as representative of the whole ToM spectrum. Along the same line, an additional spatial manipulation is present in the Other condition that could account for some of the observed EEG activation patterns. Moreover, mentalization is not a unique process and its characteristics vary substantially according to the experimental design so the present observations do not allow for drawing general conclusions about the involvement of mentalizing networks in visual perspective tasks. Nevertheless, this first complete EEG analysis could be applied in more complex paradigms to dissect the neural activation patterns needed to see the world from another person’s perspective.

## Data availability statement

The raw data supporting the conclusions of this article will be made available by the authors, without undue reservation.

## Ethics statement

The studies involving human participants were reviewed and approved by the Commission Cantonale d’Ethique de la Recherche sur l’être Humain (CCER) Swissethics. The patients/participants provided their written informed consent to participate in this study.

## Author contributions

VR, M-LM, AP, CM, and PG participated in designing the study and writing the manuscript. VR participated in conducting the analyses. VR and M-LM participated in collecting the data. M-LM and CR participated in planning participant passation and clinical evaluation. FH and AE participated in writing the manuscript. All authors contributed to the article and approved the submitted version.

## Footnotes

1
https:/sites.google.com/site/cartoolcommunity/

## References

[B1] Alcala-Lopez D. Vogeley K. Binkofski F. Bzdok D. (2019). Building blocks of social cognition: Mirror, mentalize, share? *Cortex* 118, 4–18. 29903609 10.1016/j.cortex.2018.05.006

[B2] Arora A. Schurz M. Perner J. (2017). Systematic comparison of brain imaging meta-analyses of ToM with vPT. *Biomed. Res. Int.* 2017:6875850. 10.1155/2017/6875850 28367446 PMC5359439

[B3] Bastos A. M. Vezoli J. Bosman C. A. Schoffelen J. M. Oostenveld R. Dowdall J. R. (2015). Visual areas exert feedforward and feedback influences through distinct frequency channels. *Neuron* 85 390–401. 25556836 10.1016/j.neuron.2014.12.018

[B4] Beck A. A. Rossion B. Samson D. (2018). An objective neural signature of rapid perspective taking. *Soc. Cogn. Affect. Neurosci.* 13 72–79. 10.1093/scan/nsx135 29186550 PMC5793833

[B5] Blair R. J. R. (2008). Fine cuts of empathy and the amygdala: dissociable deficits in psychopathy and autism. *Q. J. Exp. Psychol.* 61 157–170. 10.1080/17470210701508855 18038346

[B6] Brechet L. Brunet D. Birot G. Gruetter R. Michel C. M. Jorge J. (2019). Capturing the spatiotemporal dynamics of self-generated, task-initiated thoughts with EEG and fMRI. *Neuroimage* 194 82–92. 10.1016/j.neuroimage.2019.03.029 30902640

[B7] Brunet D. Murray M. M. Michel C. M. (2011). Spatiotemporal analysis of multichannel EEG: CARTOOL. *Comput. Intell. Neurosci.* 2011:813870. 10.1155/2011/813870 21253358 PMC3022183

[B8] Bukowski H. (2018). The neural correlates of visual perspective taking: a critical review. *Curr. Behav. Neurosci. Rep.* 5 189–197.

[B9] Catmur C. Santiesteban I. Conway J. R. Heyes C. Bird G. (2016). Avatars and arrows in the brain. *Neuroimage* 132 8–10. 26883064 10.1016/j.neuroimage.2016.02.021

[B10] Coetzee J. P. Monti M. M. (2018). At the core of reasoning: dissociating deductive and non-deductive load. *Hum. Brain Mapp.* 39 1850–1861. 10.1002/hbm.23979 29341386 PMC6866402

[B11] Cole G. G. Millett A. C. (2019). The closing of the theory of mind: a critique of perspective-taking. *Psychon. Bull. Rev.* 26 1787–1802. 10.3758/s13423-019-01657-y 31515733

[B12] Custo A. Van De Ville D. Wells W. M. Tomescu M. I. Brunet D. Michel C. M. (2017). Electroencephalographic resting-state networks: source localization of microstates. *Brain Connect.* 7 671–682. 28938855 10.1089/brain.2016.0476 PMC5736178

[B13] de Waal F. B. M. (2008). Putting the altruism back into altruism: the evolution of empathy. *Annu. Rev. Psychol.* 59 279–300. 17550343 10.1146/annurev.psych.59.103006.093625

[B14] Decety J. Jackson P. L. (2004). The functional architecture of human empathy. *Behav. Cogn. Neurosci. Rev.* 3 71–100. 15537986 10.1177/1534582304267187

[B15] Decety J. Moriguchi Y. (2007). The empathic brain and its dysfunction in psychiatric populations: implications for intervention across different clinical conditions. *Biopsychosoc. Med.* 1:22. 10.1186/1751-0759-1-22 18021398 PMC2206036

[B16] Delorme A. Makeig S. (2004). EEGLAB: an open source toolbox for analysis of single-trial EEG dynamics including independent component analysis. *J. Neurosci. Methods* 134 9–21. 15102499 10.1016/j.jneumeth.2003.10.009

[B17] Di Nota P. M. Chartrand J. M. Levkov G. R. Montefusco-Siegmund R. DeSouza J. F. (2017). Experience-dependent modulation of alpha and beta during action observation and motor imagery. *BMC Neurosci.* 18:28. 10.1186/s12868-017-0349-0 28264664 PMC5340035

[B18] Downing P. E. Jiang Y. Shuman M. Kanwisher N. (2001). A cortical area selective for visual processing of the human body. *Science* 293 2470–2473. 11577239 10.1126/science.1063414

[B19] Epstein R. Kanwisher N. (1998). A cortical representation of the local visual environment. *Nature* 392 598–601. 9560155 10.1038/33402

[B20] Ferguson H. J. Brunsdon V. E. A. Bradford E. E. F. (2018). Age of avatar modulates the altercentric bias in a visual perspective-taking task: ERP and behavioral evidence. *Cogn. Affect. Behav. Neurosci.* 18 1298–1319. 10.3758/s13415-018-0641-1 30242574 PMC6244738

[B21] Furlanetto T. Becchio C. Samson D. Apperly I. (2016). Altercentric interference in level 1 visual perspective taking reflects the ascription of mental states, not submentalizing. *J. Exp. Psychol. Hum. Percept. Perform.* 42 158–163. 10.1037/xhp0000138 26389611

[B22] Habermann M. Weusmann D. Stein M. Koenig T. (2018). A student’s guide to randomization statistics for multichannel event-related potentials using ragu. *Front. Neurosci.* 12:355. 10.3389/fnins.2018.00355 29973861 PMC6020783

[B23] Koenig T. Kottlow M. Stein M. Melie-Garcia L. (2011). Ragu: a free tool for the analysis of EEG and MEG event-related scalp field data using global randomization statistics. *Comput. Intell. Neurosci.* 2011:938925. 10.1155/2011/938925 21403863 PMC3049349

[B24] Kosakowski H. L. Cohen M. A. Takahashi A. Keil B. Kanwisher N. Saxe R. (2022). Selective responses to faces, scenes, and bodies in the ventral visual pathway of infants. *Curr Biol.* 32 265.e5–274.e5. 10.1016/j.cub.2021.10.064 34784506 PMC8792213

[B25] Kovacs A. M. Teglas E. Endress A. D. (2010). The social sense: susceptibility to others’ beliefs in human infants and adults. *Science* 330 1830–1834. 10.1126/science.1190792 21205671

[B26] Liegel N. Schneider D. Wascher E. Arnau S. (2022). Task prioritization modulates alpha, theta and beta EEG dynamics reflecting proactive cognitive control. *Sci. Rep.* 12:15072. 10.1038/s41598-022-19158-9 36064572 PMC9445103

[B27] Low J. Watts J. (2013). Attributing false beliefs about object identity reveals a signature blind spot in humans’ efficient mind-reading system. *Psychol. Sci.* 24 305–311. 10.1177/0956797612451469 23307943

[B28] McCleery J. P. Surtees A. D. Graham K. A. Richards J. E. Apperly I. A. (2011). The neural and cognitive time course of theory of mind. *J. Neurosci.* 31 12849–12854. 21900563 10.1523/JNEUROSCI.1392-11.2011 PMC3176738

[B29] Michel C. M. Brunet D. (2019). EEG source imaging: a practical review of the analysis steps. *Front. Neurol.* 10:325. 10.3389/fneur.2019.00325 31019487 PMC6458265

[B30] Montandon M. Rodriguez C. Herrmann F. R. Eytan A. Pegna A. J. Haller S. (2023). Patterns of multiple brain network activation in dot perspective task. *Sci. Rep.* 13:6793. 10.1038/s41598-023-33427-1 37100844 PMC10133244

[B31] Mossad S. I. Vandewouw M. M. de Villa K. Pang E. W. Taylor M. J. (2022). Characterising the spatial and oscillatory unfolding of theory of mind in adults using fMRI and MEG. *Front. Hum. Neurosci.* 16:921347. 10.3389/fnhum.2022.921347 36204717 PMC9530400

[B32] Neuper C. Pfurtscheller G. (2001). Event-related dynamics of cortical rhythms: frequency-specific features and functional correlates. *Int. J. Psychophysiol.* 43 41–58. 10.1016/s0167-8760(01)00178-7 11742684

[B33] Onishi K. H. Baillargeon R. (2005). Do 15-month-old infants understand false beliefs? *Science* 308 255–258. 15821091 10.1126/science.1107621 PMC3357322

[B34] Oostenveld R. Fries P. Maris E. Schoffelen J. M. (2011). FieldTrip: open source software for advanced analysis of MEG, EEG, and invasive electrophysiological data. *Comput. Intell. Neurosci.* 2011:156869. 10.1155/2011/156869 21253357 PMC3021840

[B35] Orgs G. Dombrowski J. H. Heil M. Jansen-Osmann P. (2008). Expertise in dance modulates alpha/beta event-related desynchronization during action observation. *Eur. J. Neurosci.* 27 3380–3384. 10.1111/j.1460-9568.2008.06271.x 18598273

[B36] Pitcher D. Charles L. Devlin J. T. Walsh V. Duchaine B. (2009). Triple dissociation of faces, bodies, and objects in extrastriate cortex. *Curr. Biol.* 19 319–324. 10.1016/j.cub.2009.01.007 19200723

[B37] Ramsey R. Hansen P. Apperly I. Samson D. (2013). Seeing it my way or your way: frontoparietal brain areas sustain viewpoint-independent perspective selection processes. *J. Cogn. Neurosci.* 25 670–684. 23249349 10.1162/jocn_a_00345

[B38] Richter C. G. Coppola R. Bressler S. L. (2018). Top-down beta oscillatory signaling conveys behavioral context in early visual cortex. *Sci. Rep.* 8:6991. 10.1038/s41598-018-25267-1 29725028 PMC5934398

[B39] Saether L. S. Roelfs D. Moberget T. Andreassen O. A. Elvsashagen T. Jonsson E. G. (2021). Exploring neurophysiological markers of visual perspective taking: methodological considerations. *Int. J. Psychophysiol.* 161 1–12. 10.1016/j.ijpsycho.2020.12.006 33388368

[B40] Samson D. Apperly I. A. Braithwaite J. J. Andrews B. J. Bodley Scott S. E. (2010). Seeing it their way: evidence for rapid and involuntary computation of what other people see. *J. Exp. Psychol. Hum. Percept. Perform.* 36 1255–1266. 10.1037/a0018729 20731512

[B41] Santiesteban I. Kaur S. Bird G. Catmur C. (2017). Attentional processes, not implicit mentalizing, mediate performance in a perspective-taking task: evidence from stimulation of the temporoparietal junction. *Neuroimage* 155 305–311. 28454821 10.1016/j.neuroimage.2017.04.055

[B42] Schneider D. Lam R. Bayliss A. P. Dux P. E. (2012). Cognitive load disrupts implicit theory-of-mind processing. *Psychol. Sci.* 23 842–847. 10.1177/0956797612439070 22760885

[B43] Schneider D. Slaughter V. P. Bayliss A. P. Dux P. E. (2013). A temporally sustained implicit theory of mind deficit in autism spectrum disorders. *Cognition* 129, 410–417. 23994318 10.1016/j.cognition.2013.08.004

[B44] Schneider D. Slaughter V. P. Dux P. E. (2017). Current evidence for automatic theory of mind processing in adults. *Cognition* 162 27–31. 28189035 10.1016/j.cognition.2017.01.018

[B45] Schurz M. Kronbichler M. Weissengruber S. Surtees A. Samson D. Perner J. (2015). Clarifying the role of theory of mind areas during visual perspective taking: issues of spontaneity and domain-specificity. *Neuroimage* 117 386–396. 10.1016/j.neuroimage.2015.04.031 25907759

[B46] Senju A. Southgate V. White S. Frith U. (2009). Mindblind eyes: an absence of spontaneous theory of mind in Asperger syndrome. *Science* 325, 883–885. 19608858 10.1126/science.1176170

[B47] Soto-Icaza P. Vargas L. Aboitiz F. Billeke P. (2019). Beta oscillations precede joint attention and correlate with mentalization in typical development and autism. *Cortex* 113 210–228. 10.1016/j.cortex.2018.12.018 30677619

[B48] Surian L. Caldi S. Sperber D. (2007). Attribution of beliefs by 13-month-old infants. *Psychol. Sci.* 18 580–586. 17614865 10.1111/j.1467-9280.2007.01943.x

[B49] Surtees A. Samson D. Apperly I. (2016). Unintentional perspective-taking calculates whether something is seen, but not how it is seen. *Cognition* 148 97–105. 10.1016/j.cognition.2015.12.010 26752604

[B50] van Kerkoerle T. Self M. W. Dagnino B. Gariel-Mathis M. A. Poort J. van der Togt C. (2014). Alpha and gamma oscillations characterize feedback and feedforward processing in monkey visual cortex. *Proc. Natl. Acad. Sci. U.S.A.* 111 14332–14341. 25205811 10.1073/pnas.1402773111 PMC4210002

[B51] Van Overwalle F. Vandekerckhove M. (2013). Implicit and explicit social mentalizing: dual processes driven by a shared neural network. *Front. Hum. Neurosci.* 7:560. 10.3389/fnhum.2013.00560 24062663 PMC3772308

[B52] Wellman H. M. Cross D. Watson J. (2001). Meta-analysis of theory-of-mind development: the truth about false belief. *Child Dev*. 72, 655–684. 11405571 10.1111/1467-8624.00304

[B53] Westra E. Terrizzi B. F. van Baal S. T. Beier J. S. Michael J. (2021). Beyond avatars and arrows: testing the mentalising and submentalising hypotheses with a novel entity paradigm. *Q. J. Exp. Psychol.* 74 1709–1723. 10.1177/17470218211007388 33752520 PMC8392802

[B54] Young L. Cushman F. Hauser M. Saxe R. (2007). The neural basis of the interaction between theory of mind and moral judgment. *Proc. Natl. Acad. Sci. U.S.A.* 104 8235–8240. 17485679 10.1073/pnas.0701408104 PMC1895935

